# A Study on Clinical and Pathologic Features in Lupus Nephritis with Mainly IgA Deposits and a Literature Review

**DOI:** 10.1155/2013/289316

**Published:** 2013-09-24

**Authors:** Liu Hongyan, Zheng Yi, Dong Bao, Lu Yuewu, Meng Juan

**Affiliations:** ^1^Department of Rheumatology, Beijing Chao-Yang Hospital, Capital Medical University, Beijing 100020, China; ^2^Department of Nephrology, Peking University People's Hospital, Beijing 100044, China

## Abstract

*Objective*. To study the clinical and pathologic features of systemic lupus erythematosus (SLE) that has atypical lupus nephritis (LN) with mainly IgA deposits. *Methods*. We searched the SLE patients who had nephritis with mainly IgA deposits in our hospital and selected the information including clinical manifestations, laboratory tests, treatments, and prognosis. *Results*. From January 2009 to June 2012, 5 patients were definitely diagnosed as SLE according to both 1982 and 2009 ACR classification criteria. But renal biopsy showed that all cases had mainly IgA deposits and were free of IgG, C1q, and fibrinogen-related antigen deposits under immunofluorescent microscopy, which did not match with typical LN. There were 2 males and 3 females, aging from 31 to 64 years and with an average of (42.20 ± 13.59) years. The 5 cases had multiple-system involvements, mainly the renal system. Compared to primary IgAN, the atypical LN showed some differences: older than primary IgAN, more women than men, no previous infection history, lower incidence of serum IgA elevation, and ACL positive rate as high as 100%. *Conclusion*. Nephritis with mainly IgAN deposits, as an atypical LN, may be a special subtype of SLE.

## 1. Introduction

In clinic, LN is divided into six types, which are minimal mesangial LN (class I), mesangial proliferative LN (class II), focal proliferative LN (class III), diffuse proliferative LN (class IV), membranous LN (class V), and advanced sclerosing LN (class VI) [1]. The previous typical LN are characterized by the so-called “Full House” stain under immunofluorescent microscopy, staining positively for IgG, IgA, IgM, C3, and C1q [2]. Besides the typical changes, some SLE patients were reported to have IgAN established by renal biopsy [3–8], staining positively for mainly IgA. Here, we report 5 cases of SLE patients who had nephritis with mainly IgA deposits.

## 2. Information and Methods

### 2.1. Patients and Information Collecting

Select the SLE patients who had nephritis with IgA-predominant deposits in our hospital from January 2009 to June 2012. The information collected included sex, age, duration, clinical and pathological manifestations, laboratory tests, treatments, and prognosis. The detailed tests were as follows. (1) Blood routine: blood leukocyte, platelet, and hemoglobin. (2) Urine routine: urine leukocyte, erythrocyte, cast. (3) 24-hour urine protein and bacteria culture of clean midstream urine. (4) Auto-antibodies: antinuclear antibody (ANA), anti-Sm antibody, anti-dsDNA antibody, anticardiolipin antibody (ACL), and anti-*β*2-glycoprotein-1 antibody (anti-*β*2-GP-1). (5) Renal biopsies examinations: HE staining, MASSON staining, PAS staining, PASM staining, and immunofluorescent staining. (6) Others: serum IgG, IgA, IgM, C3, and C4 levels and SLEDAI scores for disease activity assessment.

### 2.2. Main Reagents and Detection Methods

ANA and anti-dsDNA were both detected by indirect immunofluorescence assay, and the reagents were from German EU. Both anti-*β*2-GP-1 and ACL were tested by way of ELISA, and the ELISA kits were from German Human and German Orgenpec, respectively. Anti-Sm antibody was detected by linear immunoassay, and the reagent was from Germany Human. Serums IgA, IgG, and IgM, complements C3 and C4 were all tested by rate nephelometry, and the reagents were from the American Beckman.

### 2.3. Diagnostic Criteria

SLE was diagnosed according to both 1982 and 2009 ACR classification criteria [9, 10]. 

### 2.4. Renal Biopsy Examination

The histological changes of renal biopsies were observed by HE staining, MASSON staining, PAS staining, and PASM staining. Use the method of direct immunofluorescence to detect accumulation of IgA, IgG, IgM, complement C3, C1q, and fibrinogen-related antigen (FRA) in renal tissues, and determine the fluorescence intensity under a fluorescence microscope: “−” indicated no or weak fluorescence; “+” indicated only clearly visible fluorescence; “++” indicated bright fluorescence; “+++” indicated dazzling fluorescent.

## 3. Results

### 3.1. General Information

From January 2009 to June 2012, 5 SLE patients were established to have mainly IgA deposits by renal biopsy. In the 5 cases, 2 cases were males, and 3 were females (M/F ratio 2 : 3). The age ranged from 31 to 64 years, with an average of (42.20 ± 13.59) years. The duration lasted from 1.0 to 108 months, with an average of (29.40 ± 44.91) months. 

### 3.2. Clinical Manifestations and Laboratory Tests

In clinic manifestations, the 5 cases had multiple-system involvements, mainly renal system (5/5), manifesting in hematuria (gross hematuria 2 cases, microhematuria 3 cases), proteinuria (5/5), pyuria (2/5), cylindruria (4/5), renal dysfunction (2/5), and edema (2/5). Besides, the patients also presented hematologic involvement (3/5), serositis (2/5), and joint synovitis (1/5). In laboratory tests, anti-ANA was positive in all cases (5/5), anti-dsDNA in 3 cases (3/5), anti-Sm in 2 cases (2/5), anti-*β*2-GP-1 in 1 case (1/5), and ACL in all cases (5/5). Besides, complement C3 or C4 decreased in 4 cases (4/5), immunoglobin increased in 2 cases (2/5), and IgA increased in only 1 case (1/5). 

 In diagnosis, all cases fulfilled both 1982 and 2009 ACR classification criteria for SLE. According to 1982 criteria for SLE, cases 1 and 5 satisfied five of the criteria, and the rest cases satisfied four. According to 2009 criteria for SLE, case 1, 2, and 4 satisfied six of the criteria, case 3 satisfied five, and case 5 satisfied eight of the criteria, including at least 2 clinical criteria and 3 immunologic criteria. SLEDAI score ranged from 12 to 25, with an average of (19.2 ± 5.12). The details were as follows.

Case 1 showed gross hematuria, edema of lower limbs, pericardial effusion, and pleural effusion under ultrasound examination. The patient had no fever, cutaneous lupus, photosensitivity, oral/nasal ulcers, alopecia, inflammatory synovitis, or neurologic symptoms. Electroencephalogram was normal. Blood routine showed anemia (hemoglobin 96 g/L, normal range is 113~151 g/L) and thrombocytopenia (63 × 10^9^/L, normal 101~320 × 10^9^/L). Urine test showed hematuria, pyuria, cylindruria, and proteinuria (24-hour urine protein 2393 mg, normal 50~150 mg). Renal function was normal. ANA was positive, with the titer S1:320. ACL IgM was positive, while anti-Sm, anti-dsDNA, anti-*β*2-GP-1, ACL IgA, and IgG were negative. Complement C3 decreased (67.70 mg/dL, normal range is 79.00~152.00 mg/dL), while C4 was normal. SLEDAI score was 23. 

Case 2 had no fever, cutaneous lupus, photosensitivity, oral/nasal ulcers, alopecia, serous cavity effusion, inflammatory synovitis, or neurologic symptoms. Blood routine showed anemia (hemoglobin 98 g/L) and thrombocytopenia (81 × 10^9^/L). Urine test showed hematuria, cylindruria, and proteinuria (24-hour urine protein 2497 mg), without pyuria. Renal function was abnormal (serum creatinine was 163.00 umol/L, normal range 53.00~115.00 umol/L; serum urea nitrogen 11.10 mmol/L, normal range 2.85~7.14 mmol/L). ANA titer was S1:320, and anti-dsDNA titer was 1 : 10. ACL IgM, and IgG were positive, while anti-Sm, anti-*β*2GP-1, and ACL IgA were negative. Both C3 and C4 decreased (resp., 77.40 mg/dL and 11.00 mg/dL; normal range of C4 is 12.00~36.00 mg/dL). SLEDAI score was 17.

Case 3 had repeated oral ulcers but no fever, cutaneous lupus, photosensitivity, alopecia, serous cavity effusion, inflammatory synovitis, or neurologic symptoms. Urine test showed hematuria and proteinuria (24-hour urine protein 651 mg), without pyuria or cylindruria. Blood routine, renal function, C3, and C4 were all normal. ANA titer was S1:320, and anti-dsDNA titer was 1 : 10. Anti-*β*2GP-1, ACL IgM, and IgA were positive, while anti-Sm and ACL IgG were negative. SLEDAI score was 12.

Case 4 had hands Raynaud's phenomenon and edema of lower limbs, without fever, cutaneous lupus, photosensitivity, oral/nasal ulcers, alopecia, serous cavity effusion, inflammatory synovitis, or neurologic symptoms. Blood routine showed leukopenia (white blood cell 2.72 × 10^9^/L, normal 3.69~9.16 × 10^9^/L). Urine test showed hematuria, pyuria, cylindruria, and proteinuria (24-hour urine protein 3619 mg). Renal function was abnormal (serum creatinine 221.70 umol/L; serum urea nitrogen 28.78 mmol/L). ANA was positive, with a titer of S1:3200. Anti-Sm and ACL IgM were positive, while anti-dsDNA, anti-*β*2GP-1, ACL IgA, and IgG were negative. C3 decreased (53.10 mg/dL), while C4 was normal. SLEDAI score was 19.

Case 5 presented fever, arthritis, pericardial effusion, and pleural effusion under lung HRCT. He had no infection, cutaneous lupus, photosensitivity, oral/nasal ulcers, alopecia, or neurologic symptoms. Blood routine and renal function were normal. Urine test showed hematuria, cylindruria, and proteinuria (24-hour urine protein 4692 mg), without pyuria. ANA titer was S1:3200, and anti-dsDNA titer was 1 : 1000. Anti-Sm and ACL IgG were positive, while anti-*β*2GP-1, ACL IgA, and IgM were negative. Both C3 and C4 decreased (resp., 37.40 mg/dL and 4.50 mg/dL). SLEDAI score was 25.

Besides, all the cases had no allergic purpura, gastrointestinal, or urinary tract irritation symptoms. Bacteria cultures of clean midstream urine in all patients were negative. 3 of the 5 patients had hypertension (3/5), and 1 case had slightly abnormal coagulation (1/5; prothrombin time was 9.1 seconds, while normal value is 9.6 to 13.0 seconds; activated partial prothrombin time was 16.2 seconds, while normal value is 21 to 34 seconds; thrombin time was normal). Serum immunoglobulin was abnormal in 2 cases (2/5), and IgA increased in only 1 case (1/5). In case 1, IgG was 2340 mg/dL (normal range was 751~1560 mg/dL), IgA 1050 mg/dL (normal range was 45~382 mg/dL), and IgM 349 mg/dL (normal range was 46 to 304 mg/dL). In case 4, only IgG slightly increased (1690 mg/dL), while IgA and IgM were normal. 

Most of the previous laboratory results were listed in Tables 1 and 2.

### 3.3. Renal Biopsy Findings

**Figure 1:**
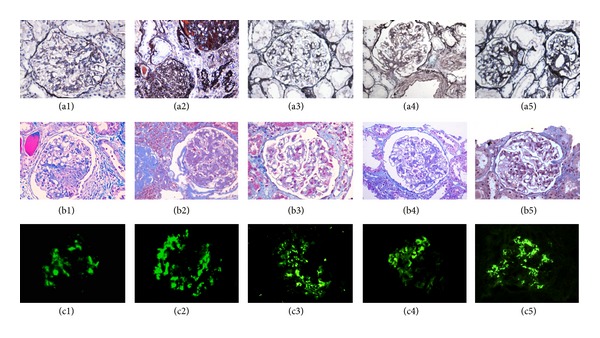
Light and immunofluorescent microscope findings of SLE that has nephritis with mainly IgA deposits. (a1), (a2), (a3), (a4), and (a5), respectively, indicate PASM staining of cases 1~5 under light microscope (×400). (b1), (b2), (b3), (b4), and (b5), respectively, indicate MASSON staining of cases 1~5 under light microscope (×400). Hyperplasia of glomerular mesangium and matrix can be seen in all cases. In addition, case 2 has clear glomerular sclerosis. (c1), (c2), (c3), (c4), and (c5), respectively, indicate IgA deposits of cases 1~5 under immunofluorescence microscope and all cases are positive (++~+++).

All cases performed light and immunofluorescent microscopy. The result showed that all cases had mainly IgA deposits and did not match with LN. Under light microscope (Figure 1), all cases showed mild diffuse hyperplasia of glomerular mesangium and matrix, with focal and segmental aggravation. The renal tubular epithelial cell showed vacuolar degeneration, granular degeneration, and spotty or flake atrophy, while the renal interstitial showed fibrosis and infiltration of lymphocytes and monocytes. In case 2, glomerular sclerosis can be clearly seen. Immune complex deposits were seen in glomerular mesangium under immunofluorescent microscope. All cases had IgA deposits (Figure 1) and were free of IgG, C1q, and FRA deposits. In addition, as well as IgA deposit, 1 case had C3 deposit, and the other 4 cases had IgM and C3 deposits.

### 3.4. Treatment and Prognosis

All cases were given prednisone at a dose of 1 mg/(kg·d) after percutaneous renopuncture, and cases 1, 2, and 5 also received intravenous cyclophosphamide treatment. All the cases achieved remission after therapy, for example, clinical symptoms got relief (such as arthritis, edema, orrhomeningitis, Raynaud's phenomenon, and oral ulcers), blood routine, urine tests, and immunological tests improved, including reduction of protein, red blood cells, white blood cells, and casts in urine, decrease of SLEDAI score, as well as increase of white blood cells, platelet, C3, and C4.

## 4. Discussion

### 4.1. Diagnosis of SLE

In 1982 ACR classification criteria for SLE, if the patient satisfies four or more than four of the criteria, we can classify the patient as having SLE. According to that, cases 1 and 5 satisfied five of the criteria, and the rest cases satisfied four. So they can be definitely diagnosed as SLE. In 2009 ACR classification criteria for SLE, if (1) the patient has biopsy-proven LN with ANA or anti-dsDNA or (2) the patient satisfied four of the criteria, including at least one clinical and one immunologic criterion, we classify the patient as having SLE. The 5 patients in our study were in the second case. They satisfied 5 to 8 criteria, including at least 2 clinical criteria and 3 immunologic criteria. Even if we exclude renal injury, the patients still satisfied 4 to 7 criteria and can be diagnosed as SLE. So, whichever criteria we choose or whether we include renal injury, the five cases can be diagnosed as SLE. 

Typical LN are characterized by “Full House” stain under immunofluorescent microscopy, staining positively for IgG, IgA, IgM, C3, and C1q. However, the five SLE patients showed mainly IgA deposits and free of IgG and C1q deposits, which did not match with typical LN. It is unusual in a clinic for SLE patients to have nephritis with mainly IgA deposits, so we made a review to get a further understanding of the problem. 

### 4.2. Relationship between SLE and Nephritis with Mainly IgA Deposits

In the recent 3.5 years, as many as 5 SLE patients in our study were found to have mainly IgA deposits by renal biopsy. Retrieved from the Pubmed and Chinese National Knowledge Infrastructure (CKNI) since 1995, 8 pieces of literature reported a total of 10 SLE patients who have nephritis with mainly IgA deposits, all of whom were regarded as IgAN [3–8]. In these pieces of literature, the relationship between SLE and IgAN was discussed, and the result is still controversial. Most scholars believe that the typical LN includes the previous 6 types but not IgAN. They regard IgAN as a complication of SLE when the two diseases coexist [3, 4, 7], which means that the patient is affected by SLE and IgAN simultaneously. In typical LN, the complements C1q, C3, and C4 and the IgG-predominant accumulation of polyclonal immune complexes can be seen in the capillary basal membrane. In IgAN, however, IgA is the major deposit. At the same time, 22–60% of IgAN patients combine with IgM deposit, and 80% combine with C3 deposit. The previous features of IgAN do not match with the typical LN.

Some other scholars believe that IgAN may be a special clinical subtype of SLE. In 2010, the Japanese scholar Horino et al. [6] reported a case of a male SLE patient whose renal biopsy was established as class II LN. He was given the second renal biopsy because of repeated proteinuria, and the result suggested IgAN. The authors proposed that IgAN may be a special clinical subtype of SLE. As there is a mutual transition among the types of typical LN, typical LN may also convert into IgAN.

In our study, we found some differences in clinical characteristics between atypical LN with mainly IgA deposits and primary IgAN. The primary IgAN mainly occurred in young men, mostly 20 to 30 years old. Before the onset of IgAN, the patients can affect upper respiratory or gastrointestinal infection. Primary IgAN is characterized by gross hematuria or asymptomatic microscopic. They may also have edema, hypertension, renal dysfunction, and other clinical manifestations. Besides, serum IgA level is higher than normal in about 50% patients. The five cases in our report, similar to primary IgAN, showed gross hematuria or microscopic hematuria, edema, hypertension, renal dysfunction, and so on. However, compared with primary IgAN, the five patients have some different characteristics: older than primary IgAN, more women than men, no previous infection history before disease onset, and low incidence of serum IgA elevation. Some SLE patients are ACL positive, and the rates reported are different but not more than 60% [11]. However, the ACL positive rate in this report is as high as 100%. ACL can interfere with blood clotting mechanism, resulting in generation and aggravation of hypercoagulable state. It can also cause coagulation in the glomerular capillary and aggravate kidney damage. This may be one of the reasons why the five cases of SLE patients have much more serious kidney damage. However, it is still unclear why the ACL positive rate increases in SLE that had nephritis with mainly IgA deposits.

We speculate that nephritis with mainly IgA deposits may be a special clinical subtype of SLE. In other words, nephritis with mainly IgA deposits, as an atypical LN, may be another nephropathy of SLE in addition to the typical LN. The supporting points are as follows. (1) The cases of nephritis with mainly IgA deposits are not rare. As many as 10 cases have already been reported before, and other 5 cases are found here in only 3.5 years. Also, it is possible that some similar cases were not reported because of neglect. (2) There was no significant difference between SLE with typical LN and SLE with atypical LN in clinical manifestations or laboratory tests. (3) Compared with primary IgAN, nephritis with mainly IgA deposits observed in SLE has its own clinical characteristics as mentioned previously: older than primary IgAN, more women than men, low incidence of serum IgA elevation, and ACL positive rate as high as 100%. To conclude, nephritis with mainly IgA deposits, as an atypical LN, may be a special clinical subtype of SLE, although it still needs a lot of further research.

### 4.3. Pathogenesis

SLE is also an immune-complex-mediated disease. Although C1q, C3, C4, and IgG depositions [2] are more common in typical LN, IgA deposition can also be seen. We speculated that, when some uncertain mechanisms lead to a majority of IgA deposition, the renal biopsy finding may present atypical LN with mainly IgA deposits.

### 4.4. Prognosis

In this research, five patients got relief after treatment of glucocorticoids and immunosuppressive drugs. The relief indicates good effect of glucocorticoids and immunosuppressive drugs on SLE that has atypical LN with maily IgA deposits. But there are also some complex and ineffective cases reported. Lai et al. [8] reported one patient who died of systemic infection. This alerts clinicians that they should give early diagnosis and treatment to such patients and be aware of the appearance of complications, especially infection.

In summary, nephritis with maily IgAN deposits is not rare in SLE and has its own clinical characteristics which are different from those of primary IgAN. We speculated that nephritis with mainly IgAN deposits, as an atypical LN, may be a special subtype of SLE. It may be another nephropathy of SLE in addition to the typical LN and has a relative good prognosis. However, the speculation still needs further clinical observation and research.

## Figures and Tables

**Table 1:** Laboratory test results of SLE that has nephritis with mainly IgA deposits.

Case	HGB (g/L)	WBC (×10^9^/L)	PLT (×10^9^/L)	ANA	dsDNA	Sm	C3 (mg/dL)	C4 (mg/dL)	Anti-*β*2 GP-1	ACL	SLEDAI score
1	96^▲^	5.22	63^▲^	1 : 320	−	−	67.70^▲^	16.30	−	IgM(+)	23
2	98^▲^	6.41	81^▲^	1 : 320	1 : 10	−	77.40^▲^	11.00^▲^	−	IgM, G(+)	17
3	124	5.35	279	1 : 320	1 : 10	−	104.00	14.70	+	IgM, A(+)	10
4	145	2.72^▲^	237	1 : 3200	−	+	53.10^▲^	13.10	−	IgM(+)	19
5	124	9.82	214	1 : 3200	1 : 1000	+	37.40^▲^	4.50^▲^	−	IgG(+)	25

^▲^Indicates lower than normal; + indicates positive; − indicates negative. Normal range: (hemoglobin) HGB 113~151 g/L; (white blood cells) WBC 3.69~9.16 × 10^9^/L; (platelet) PLT 101~320 × 10^9^/L; C3 79~152 mg/dL; C4 12.0~36 mg/dL.

**Table 2:** Renal involvement of SLE that has nephritis with mainly IgA deposits.

Case	Sex age	Red cells (per HPF)	White cells (per HPF)	Casts (per HPF)	Protein (g/24 h)	Serum creatinine (umol/L)	Serum urea nitrogen (mmol/L)
1	F/64	All view	All view	8–10 granular	2393^▲^	74.60	4.49
2	M/45	All view	—	1-2 granular	2497^▲^	163.00^▲^	11.10^▲^
3	F/31	30–35	—	—	651^▲^	60.60	4.00
4	F/31	24–26	20-21	1–3 granular	3619^▲^	221.70^▲^	28.78^▲^
5	M/40	15–20	1-2	1–3 granular	4692^▲^	86.10	5.22

^▲^Indicates higher than normal range. Red cells, white cells, cast, and protein were all tested in urine. Normal range: urine protein (g/24 h): 50~150 mg; serum creatinine 53~115 umol/L; serum urea nitrogen 2.85~7.14 mmol/L.
